# Cardiovascular and Cerebrovascular Morbidity in Patients with Urolithiasis: An Epidemiological Approach Based on Hospitalization Burden Data from 1997 to 2021

**DOI:** 10.3390/jcm13123564

**Published:** 2024-06-18

**Authors:** Javier Sáenz-Medina, Victoria Gómez Dos Santos, María Rodríguez-Monsalve, Alfonso Muriel-García, Manuel Durán-Poveda, Alfonso Gómez del Val, Javier Burgos Revilla, Dolores Prieto

**Affiliations:** 1 Department of Urology, Puerta de Hierro-Majadahonda University Hospital, Calle Manuel de Falla, 1, 28222 Majadahonda, Spain; mrmonsalveherrero@salud.madrid.org; 2 Department of Medical Specialties and Public Health, King Juan Carlos University, 28922 Madrid, Spain; manuel.duran@hospitalreyjuancarlos.es (M.D.-P.); dprieto@ucm.es (D.P.); 3 Department of Urology, Ramón y Cajal University Hospital, 28034 Madrid, Spain; vgomezd@salud.madrid.org (V.G.D.S.); fjavier.burgos@salud.madrid.org (J.B.R.); 4 Department of Clinical Biostatistics, Ramón y Cajal University Hospital, 28034 Madrid, Spain; alfonso.muriel@uah.es; 5 Department of Physiology, Pharmacy Faculty, Complutense University, 28040 Madrid, Spain; alfonsovaldelgomez@gmail.com

**Keywords:** urolithiasis, coronary heart disease, cerebrovascular disease, endothelial dysfunction, epidemiology

## Abstract

**Background:** Patients with kidney stones (KSFs) are known to have a heightened risk of coronary heart disease (CHD) or stroke. The objective of the present study was to describe the natural history of these complications through the longitudinal analysis of the hospitalizations due to kidney stones in Spain from 1997 to 2021. **Methods**: A retrospective longitudinal observational study was developed based on nationwide hospitalization data (minimum basic data base). Three different analyses were carried out. In the first step, the prevalence of coronary or cerebrovascular events in kidney stone hospitalizations was compared with the hospitalization burden of CHD or strokes related to the general population. In the second step, a survival analysis of the kidney stones–hospitalized patients using the Kaplan–Meier method was conducted. In the third step, a Cox regression was used to assess the influence of the classical comorbidities in the development of the lithiasic patients–cardiovascular disease. **Results**: Kidney stone-hospitalized patients exhibit a significantly higher risk of CHD (OR = 14.8 CI95%: 14.7–14.9) and stroke (OR = 6.7 CI95%: 6.6–6.8) compared to the general population across in all age groups, although they had less cardiovascular risk factors. A total of 9352 KSFs (1.5%) developed a coronary event within an average time of 78.8 months. A total of 2120 KSFs (0.33%) suffered a stroke in an average time of 71.1 months. Diabetes, hypertension, hyperlipidemia, and being overweight were identified as risk factors for developing CHD and stroke using a univariate and multivariate analysis. **Conclusions**: Our study confirms previous studies in which kidney stones must be considered as a risk factor for developing CHD or cerebrovascular disease. Preventive strategies should target patients with kidney stones and classical risk cardiovascular factors to mitigate modifiable conditions associated with cardiovascular diseases.

## 1. Introduction

An increased risk of cardiovascular disease (CVD), myocardial infarction, and stroke in patients with kidney stones has been widely reported. To date, four metanalyses have examined the association between kidney stones cardiovascular and cerebrovascular disease. Coronary heart disease (CHD) relative risk specifically associated with kidney stones has been established to be between 1.20 and 1.25. Similarity, stroke has also been correlated with urolithiasis, presenting relative risks ranging from 1.17 to 1.40 [1,2,3,4].

The studies included in the metanalyses previously mentioned assessed CHD in patients with kidney stones, with the most common outcome in this category being acute myocardial infarction (MI). Some of them were longitudinal cohort studies in which patients with urinary calculi were compared with a control group, including large samples and collected data from national health insurance databases. Ferraro et al. [5] extracted data from three health professional follow-up studies in which self-reported patients with a history of kidney stones were compared with patients without a history of urolithiasis. Studies concerning cerebrovascular disease assessed strokes/transient ischemic attacks (TIA), revealing a statistically significantly higher risk of stroke in kidney stone formers (KSFs). No survival analysis has been found in any of them, and no data of incidence has been reported up to date.

Several links have been pointed out as responsible for the relationship between urolithiasis and CVD. The impact of kidney stones on renal function and the associated deterioration is a cause of increased CVD, with a possible common link [2]. Oxidative stress has also been reported as a common underlying mechanism between low-chronic-inflammation diseases such as diabetes, obesity, or kidney stones and CVD [6,7,8]. Endothelial dysfunction (ED), characterized by impaired vasodilator response, altered angiogenesis, and barrier function, along with overexpression of pro-inflammatory and pro-thrombotic factors, is considered to be the initial stage of atherosclerosis vascular disorders [9,10]. Oxidative stress (OS) has been recognized as a key factor for ED development. Studies conducted by our group have shown, in an animal model of hyperoxaluria, how hyperoxaluria is associated with altered endothelium-dependent relaxing responses in kidney preglomerular arteries mediated by reactive oxygen species (ROS) [11].

No large analysis describing the relationship between urolithiasis and CVD based on a survival analysis has been reported. Consequently, the incidence of CHD and stroke in patients with kidney stones and whether kidney stones can be considered an independent risk factor for these pathologies remain undetermined. In this study, we aimed to establish the incidence of CHD and stroke in patients with kidney stones. For this purpose, we have analyzed hospitality burden data from minimum basic data system (MDBS) available in Spain from 1997 to 2021.

## 2. Methods

### 2.1. Study Design

A retrospective longitudinal observational population-based study was conducted based on MDBS, which collects about 98% of the hospitalizations nationwide, both for public and private hospitals. The data were collected from 1 January 1997 to 31 December 2021.

All patients were identified by a medical record number and were selected if the main or secondary diagnosis code was the diagnosis of urolithiasis, CHD, or cerebrovascular disease. The codes collected were based on the ninth edition of the International Classification of Diseases (ICD-9-MC) from 1997 to 2015 and the tenth edition (ICD-10-MC) from 2015 to 2021 (see Table 1). Additional variables such as age, gender, secondary diagnosis (up to 14 additional diagnosis codes), hospitalization length, cost of hospitalization, and discharge destination were also collected. The rates of hospitalization were related to the general population reported by the National Institute of Statistics (NIS) and were calculated per 100,000 inhabitants [12].

### 2.2. Statistical Analysis

MDBS is an administrative database based on hospital discharge reports. It is mandatory for all public and private hospitals, so it covers 95–97% of hospitalizations in Spain [13]. These data, related to the general population, can be considered as the total burden hospitalizations per 1,000,000 inhabitants. The comparison of the cardiovascular and cerebrovascular morbidity in the patients with kidney stones with the total burden hospitalization of these two diagnoses will serve us to quantify the influence of the stone disease as a risk factor for the development of a cardiac ischemic or cerebrovascular event. A comparison was performed using a chi square test. Odds ratios were also calculated to determinate the magnitude of the difference.

In a second analysis, a longitudinal follow-up and a survival analysis was developed with the patients who were hospitalized for kidney stones using the Kaplan–Meier method in order to determine the time elapsed between the occurrence of the risk factor (renal/ureteral stone) and the subsequent cardiovascular or cerebrovascular event. A univariable analysis was also performed in order to calculate the hazard ratios and log-rank test of sex and classical cardiovascular risk factors and to know if they were maintained in patients with kidney stones. A multivariable analysis was also performed using Cox regression to evaluate the real influence of each risk factor in the presence of the others.

Statistical differences were achieved when *p* < 0.05 and 95% confidence intervals were calculated both for rates (hospitalization burden) and for odds ratios. SPSS 21.0 and Excel 2016 were used for developing statistical calculations.

## 3. Results

### 3.1. Demographic and Basal Characteristics

The total Spanish hospitalizations collected by the MDBS, coded as urolithiasis or coronary heart or cerebrovascular diseases, since 1997 to 2021 were analyzed separately. A total of almost 7 million hospitalizations were analyzed. CHD was the widest group, with almost 4.5 million hospitalizations, followed by the cerebrovascular disease group with 1.3 million, and over 1 million hospitalizations of patients with kidney stones. Detailed figures are provided in Table 2.

The CHD group had a higher proportion of male patients compared to the other groups. These patients, along with the cerebrovascular disease patients, were also older than the patients with kidney stones and presented a significantly higher prevalence of cardiovascular risk factors (diabetes, hypertension, hyperlipidemia, being overweight, and smoking).

The hospitalizations of patients with kidney stones predominantly involved the youngest patients, presenting the lowest proportion of diabetes, hypertension, hyperlipidemia and obesity, also a significantly lower proportion of smoking than the CHD group.

Cerebrovascular disease hospitalizations occurred in the oldest patients. This group presented the highest proportion of hypertension and the lowest proportion of smoking codes, in contrast with CHD patients, who presented the highest proportion of people who smoked (5.3%). All the data are presented in Table 2.

### 3.2. Cardiovascular and Cerebrovascular Events in General and in Patients with Kidney Stones

As shown in Table 3, stone disease multiplies the possibilities of suffering CHD compared to the general population by 14.8. This circumstance is more evident in youngest population, in which urolithiasis multiplies the possibilities of suffering CHD morbidity by 26.3, in contrast with the oldest population in which the odds ratio is 3.4.

Cerebrovascular morbidity followed the same pattern as CHD, although the association was slightly lower. Again, stone disease multiplied the probability of suffering a stroke in the overall population by 6.7. In the youngest population, the odds ratio was 11.6, while in the oldest population, it was 1.6. With increasing age, the odds ratio decreased inversely. The statistical significance was maintained in every group of age (*p* > 0.0001). Therefore, although the patients with kidney stones presented a lower risk of suffering a stroke than an ischemic cardiac event, in both cases it, followed the same pattern, likely because both malignancies share ED as a previous stage risk factor.

### 3.3. Survival Analysis

As a second analysis, a longitudinal follow-up was developed for lithiasic hospitalizations, since a medical record number identified each patient and the hospitalizations were collected from 1997 to 2021.

**Figure 1:**
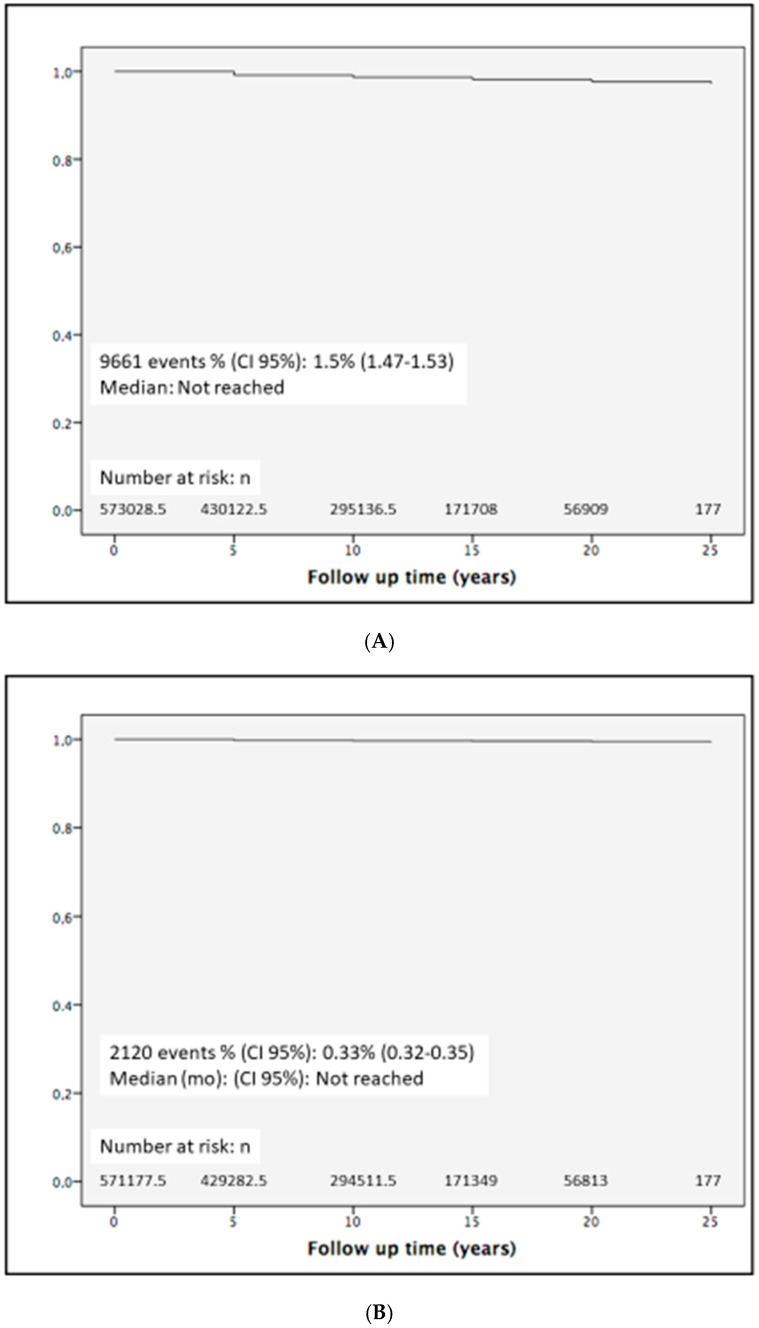
Kaplan–Meier curves for ischemic heart (**A**) and cerebrovascular disease occurrence (**B**) in patients with kidney stones.

The patients’ demographic characteristics and cardiovascular risk factors, coded as comorbidities, are shown in Table 4. Among the 1,070,192 hospitalizations, data from 642,207 patients were collected. The mean follow-up was 140.9 months (CI95%: 140.7–141.1) months. During the 300 months of follow-up (1997–2021), 9352 KSFs (1.5% CI95% 1.5–1.5) suffered a coronary event. The mean time to the cardiac event was 78.8 months (CI95%: 77.4–80.2), with a median time of 59 months. Concerning cerebrovascular disease, 2120 KSFs (0.33% CI95% 0.32–0.34) suffered a stroke. The mean time to the stroke was 71.1 months (CI95% 68–74.2), with a median time of 46 months. The Kaplan–Meier curves are detailed in Figure 1.

The demographic and clinical features stratified by major adverse cardiovascular events (MACE) are shown in Table 4. The patients with kidney stones with CHD or stroke were older and presented a significant higher mortality than the patients without events. Mortality rates were notably elevated among patients with CHD (12.1%) and stroke (18.3%) compared to those without MACEs (4.5%). The detailed distribution of cardiovascular risk factors, stratified by the different groups of patients, is represented in Table 4.

### 3.4. Univariate and Multivariate Cardiovascular Risk Factor Analysis

To complete the study, classical cardiovascular risk factors were tested in order to analyze their influence for developing CHD or stroke in patients with kidney stones. A univariable analysis showed how females presented a higher risk of CHD, in contrast to cerebrovascular disease, in which no differences were found between either sex. The remaining classical cardiovascular risk factors except for smoking (diabetes, hypertension, hyperlipidemia, being overweight, and smoking) were found as risk factors for developing both coronary and cerebrovascular events. Diabetes showed the highest HR, multiplying the possibility of developing a cerebrovascular event by 2.97 or a coronary event by 1.81. Every risk factor showed a stronger influence for developing cerebrovascular events than CHD, except for hyperlipidemia, in with both HRs were very similar. The data are shown in Table 5.

Every risk factor except for smoking remained significant in the presence of the others when a multivariable analysis was performed using Cox regression. Diabetes was the most important risk factor both for developing CHD and cerebrovascular events, and it showed a slightly higher influence for developing cerebrovascular events than CHD. The data are shown in Table 6.

## 4. Discussion

Renal stone disease has been widely recognized as a chronic low-inflammatory disease with systemic effects. It is frequently associated with systemic metabolic disease such as diabetes, obesity, or metabolic syndrome [14,15,16]. Moreover, the increased prevalence observed in recent decades has been linked to the rising rates of obesity and being overweight that have developed in industrialized countries [17,18].

Oxidative stress has been pointed out as a pathogenic factor involved in chronic low-inflammatory states such as diabetes, metabolic syndrome, obesity, or stone disease. Several approaches in vivo [6] and ex vivo [19] have investigated the impact of oxidative stress on kidney stone formation. Additionally, kidney stones have been associated with other metabolic injuries such as diabetes, being overweight, or metabolic syndrome [11]. Oxidative stress has been implicated in the mechanisms of stone formation in which endothelial injury developed [20]. In addition to oxidative stress induced by tubular injury, it has been pointed out that the action of oxidative stress occurs over the vascular bed, not only in the kidney but also in the systemic vascular system. Endothelial dysfunction has been recognized as an oxidative stress-induced first step for developing atherosclerosis and therefore CVD [9,10]. Furthermore, oxidative stress contributes to renal tubular cell apoptosis and atherosclerosis, further exacerbating endothelial dysfunction [21]. The apoptotic process in the vascular smooth muscle cells within atherosclerosis plaques and intimal calcification is associated with the expression of osteopontin, a mediator of kidney stone formation [22].

Experimental and clinical approaches have also shown that endothelial dysfunction is persistently developed in patients with kidney stones and in various experimental models [11,21,23,24]. A slight relationship between the stone disease and the development of CVD and cerebrovascular disease in which hazard ratios range between 1.1 and 1.5 has also been widely shown using different epidemiological approaches [1,2,3,4]. However, no earlier studies have been developed based on large national registries.

In the present study, we conducted a longitudinal approach based on the Spanish national registry of hospitalizations from 1997 to 2021. The extensive sample size and the 25-year follow-up period have enabled us to elucidate the natural history of how stone disease precipitates cardiovascular and/or cerebrovascular events.

As far as we know, no studies have been developed for this purpose based on this design. In order to quantify the influence of the stone disease as a risk factor, we compared the incidence of CHD or stroke in patients with kidney stones with that of general population. Hospitalization burden data related to the general population can be used as a similar measure of prevalence, since Spanish MDBS covers almost 100% of the hospitality discharges, and the vast majority of people with these pathologies seek treatment at hospital emergency departments.

As shown in Table 3, the prevalence of CHD in lithiasic hospitalization discharges was 14.8 times higher than the CHD-coded diagnosis hospitalization burden related to the general population. This odds ratio became higher as the age became lower; therefore, although the prevalence of CHD is significantly higher in patients with kidney stones compared to the general population, this difference is much more important in young people. Therefore, according to these data, kidney stones constitute a relevant cardiovascular risk factor, especially for young people, in which it seems to exert a greater influence than classical cardiovascular-related threats.

There are no data about CHD hospitalization burden prevalence related to the Spanish general population based on MDBS. Estimating the true prevalence of CHD in the general population is challenging due to considerable variability in the terminology, definitions, and criteria used across studies and official statistics when assessing its impact. Most information on coronary morbidity and mortality is drawn from data provided by national surveys and observational cohort studies [25]. Although there are no consistent studies about the true prevalence of CHD in Spain, a prevalence of angina in the general population of 7.3% in men and 7.7% in women has been reported [26]. Data obtained from official statistics of the European Union on CVD set the rate of CHD hospital discharges from 1995 to 2009 between 278 and 365 patients per 100,000 inhabitants [27]. This range aligns closely with our study, which estimates a rate of CHD discharges of 362.9 patients per 100,000 inhabitants between 1997 and 2021.

Our data show a higher influence of the stone disease on the CHD development compared to the data reported so far, in which the relative risk of developing CHD is slightly higher than the general population (RR: 1.2–1.25). Four metanalyses have been reported so far [1,2,3,4]. The most recent one includes eight studies that report a significant relationship between kidney stones and CHD, although substantial heterogeneity was detected. This heterogeneity can be attributed to various factors such as sex, geographic region, and duration of follow-up.

The disparities observed between our study and previous research can be attributed to several factors. First, our study is based on hospitality burden data in which the CHD prevalence of patients with kidney stones is compared with general population data. Consequently, the severity of stone disease and comorbidity may be higher in our study compared to the studies included in metanalysis in which many patients may not have experienced complications related to kidney stones or have reported comorbidities. Additionally, those studies were compared with a control group. In our study, the prevalence of CHD in KSFs was compared with hospitalized patients for CHD, assuming that all patients with CHD have been hospitalized. On the other hand, all groups of age were included, in contrast with most of the studies included in the metanalysis where only adult patients are analyzed. The youngest patients with kidney stones showed the highest risk of developing CHD, and this circumstance also could magnify the differences. Moreover, our study featured a longer follow-up compared to previous research where follow-up periods typically ranged from 10 to 13 years. For all these reasons, the design of our study could enhance the differences described in the previous studies.

Similar results have been obtained when analyzing the incidence of stroke in patients with kidney stones. Stone patients presented a 6.7 times greater risk of suffering a stroke compared to stroke-hospitalized patients in the general population. Eight papers based on the results were included in the last metanalysis regarding this issue. The pooled results indicate that having a history of kidney stones may increase the risk of stroke, with a relative risk of 1.17 [4]. Again, the hospitalization conditions of our patients, the design of our study, the different groups of age included, and a longer follow-up period could explain the higher risk found when analyzing our data.

Strikingly, in Table 2, it is shown that the CHD and cerebrovascular disease population have significantly more cardiovascular risk factors than KSFs, thus enhancing the fact that urolithiasis is a risk factor for developing CHD and stroke.

To our knowledge, no studies have previously described the natural history of CHD or stroke in patients with kidney stones. In this study, 1.5% of KSFs developed CHD, and 0.33% developed a stroke. Determining the exact prevalence of CHD or stroke from the different studies can be challenging. Xu [28] reports a CVD prevalence of 4.02% and a CHD prevalence of 1.44%, which is very close to ours. However, a 2.7% stroke prevalence was reported, which is significantly higher than our data. From Hsu’s study [29], it is possible to extract a 1.8% of stroke prevalence, which is also higher than ours.

This is the first study that reports the natural history of CHD and stroke in KSFs using the Kaplan–Meier method. A small proportion of KSFs suffer a cardiac event, taking an average time of 78.8 months. Concerning lithiasic patients that suffer a stroke, it takes an average time of 71.1 months. No studies have been carried out to characterize CHD or stroke in patients with kidney stones based on the Kaplan–Meier method. Furthermore, only a few studies [30,31] have been found to report the incidence of CHD or stroke based on Kaplan–Meier curves, and none of them were based on large population cohorts, using different methodological settings, making it challenging to contrast our results. Nonetheless, when comparing our survival curves with different populations previously reported with similar designs [30], it can be foreseen how the pattern followed by our stone patients is comparable with those who present a moderate risk of major adverse cardiovascular events.

Once the lithiasis condition has been established as a risk cardiovascular factor, identification and evaluation of the classical comorbidities is critical for disease prevention and to implement treatment strategies. In fact, the influence of common risk factors such as obesity, diabetes [15], hyperlipidemia [32], or hypertension [33] could underlie this connection. In this study, it has been shown how females show a higher risk of CHD. This feature has already been described by other metanalyses in which it has been showed that the association between urolithiasis and coronary artery disease was higher in women (pooled HR = 1.43) compared to men (pooled HR = 1.19) [4], as well as a higher risk of suffering MI (RR = 1.37) [2]. Conversely, the differences between sexes were not significant when considering cerebrovascular disease, contrasting with a previous analysis that reported a slightly higher prevalence of stroke in women (RR = 1.12) [2] (HR = 1.2) [4].

According to our data, diabetes, hypertension, hyperlipidemia, and being overweight have been shown to be risk factors for CHD and stroke in stone patients, as demonstrated using both univariate and multivariate analyses. It is remarkable that, concerning metabolic diseases, although the prevalence in the different groups was higher for the CHD and stroke groups (Table 4), these differences were enhanced when calculating hazard ratios. As mentioned above, metabolic disorders have extensively been reported to be common risk factors for kidney stone formation and CVD. Recent studies have demonstrated that endothelial dysfunction is a common feature developed by these malignancies, and it has also been pointed out also as a mechanism that causes urolithiasis [34,35]. Oxidative stress underlies endothelial dysfunction and has been involve in both CVD [7,8] and kidney stone formation [36,37]. Therefore, metabolic diseases might doubly contribute to the pathogenesis of CVD in KSFs, on the one hand as a promoter of urolithiasis-induced oxidative stress and, on the other, as a classical CVD risk factor. Thus, special attention must be paid to patients with kidney stones and these disorders because CHD, stroke, and mortality are clearly enhanced, and preventive strategies must be implemented in order to revert endothelial dysfunction [37].

This study has several strengths, including the large nationwide sample size, the longitudinal design, and the fact that MDBS collects 99% of the hospitalizations in Spain. However, it should be noted that the present data cannot be utilized to calculate the prevalence of kidney stones in Spain, as many cases may be asymptomatic and thus underestimated.

On the other hand, the information collected is all validated, since it comes from the physician’s diagnosis, unlike several studies based on self-reported interviews. However, no relationship can be inferred with the different types of kidney stones, since that information is not collected either by ICD-9-MC or by ICD-10-MC. In Spain, it has been reported that the most common types are made from calcium, followed by uric acid, struvite, or cystine [38].

Regarding CHD and stroke, in our environment, the vast majority of symptomatic MACEs are controlled by the emergency departments and are coded and included in the MDBS dataset. Nevertheless, this amount could be underestimated, since some of them could be asymptomatic and not detected as a personal antecedent in the hospitalization clinical records.

In conclusion, our study confirms previous studies in which kidney stones are considered as a risk factor for CHD and stroke. This confirmation is achieved through a novel approach based on a longitudinal design over a large nation-wide dataset. These associations are particularly significant in younger individuals and take place within an average timeframe of 6 to 7 years following the diagnosis of urolithiasis. Patient with lithiasis and/or metabolic disorders must be specially considered, since they develop a greater risk of MACEs and death. Preventive strategies should be tailored to address modifiable conditions associated with endothelial dysfunction and kidney stones in these patients.

## Figures and Tables

**Table 1:** ICD 9 and 10 codes of kidney or ureteral stone, cardiovascular, and cerebrovascular events.

	CIE9 MC	CIE10 MC
Description	1997–2016	2016–2021
Diagnostic Codes		
Ureteral stone	592.1	N20.1
Kidney stone	592.0	N20.0
Kidney and ureteral stone		N20.2
Hydronephrosis with renal and ureteral stone obstruction	591	N13.2
Non specified urinary stone	592.9	N20.9
Angina pectoris	413.9	I20
Acute heart attack	410/410.9	I21
Cardiac chronic ischemic disease	414.9	I25
Cerebral stroke and cerebrovascular disease	434	I63/I67

**Table 2:** Demographic factors and cardiovascular risk factors of the three selected populations (urolithiasis, coronary heart disease, or cerebrovascular disease). Continuous variable data (age) are given as mean and 95% CI (CI95%). Categorical variables are given as percentage of first category and 95% CI.

	Patients with Kidney Stones	Coronary Heart Disease	Cerebrovascular Disease	*p*
n (Hospitalizations)	1,070,192	4,495,002	1,263,321	*p* < 0.0001
Gender (M/F)	55.5 (55.4–55.6)	67.8 (67.8–67.9)	51.8 (51.7–51.9)	*p* < 0.0001
Mean Age (years)	57.9 (57.9–58)	72.2 (72.2–72.2)	74.3 (74.3–74.3)	*p* < 0.0001
Diabetes (%)	4.9 (4.9–4.9)	16.7 (16.7–16.8)	8.4 (8.4–8.5)	*p* < 0.0001
Hypertension (%)	21.2 (21.1–21.2)	42.0 (42.0–42.0)	43.9 (43.8–43.9)	*p* < 0.0001
Hyperlipidemia (%)	7.1 (7.0–7.1)	16.3 (16.2–16.3)	15.6 (15.6–15.7)	*p* < 0.0001
Overweight (%)	4.3 (4.3–4.4)	12.0 (12.0–12.0)	5.6 (5.5–5.6)	*p* < 0.0001
Smoking (%)	2.9 (2.8–2.9)	5.3 (5.3–5.4)	2.5 (2.5–2.6)	*p* < 0.0001

**Table 3:** Hospitalization rates (rate per 100,000 inhabitants) of cardiovascular and cerebrovascular diagnosis related to the general population and to lithiasic hospitalizations in Spain by age (1997–2021). Data are given as rate (95% CI) per 100,000 inhabitants. All odds ratios reached statistical significance (*p* < 0.0001).

Age	Cardiovascular Morbidity in General Population	Cardiovascular Morbidity in Lithiasic Population	Odds Ratio (*p*)	Cerebrovascular Morbidity in General Population	Cerebrovascular Morbidity in Lithiasic Population	Odds Ratio (*p*)
<20 years	0.6 (0.55–0.61)	15.12 (4.9–35.3)	26.3 (10.9–63.2)	1.83 (1.77–1.83)	21.2 (8.51–43.63)	11.6 (5.5–24.3)
20–29 years	2.0 (1.9–2.0)	29.3 (15.6–50.1)	14.8 (8.6–25.6)	2.4 (2.3–2.4)	20.3 (9.3–8.5)	8.6 (4.5–16.6), *p* < 0.0001
30–39 years	17.4 (17.2–17.6)	133.3 (111.7–157.8)	7.7 (6.5–9.1)	6.7 (6.6–6.8)	40.8 (29.3–55.3)	6.1 (4.5–8.2)
40–49 years	104.5 (104.1–105)	951.2 (902.3–1002)	9.2 (8.7–9.7)	22.7 (22.5–23)	119.1 (102.1–138)	5.2 (4.5–6.1)
50–59 years	331.5 (330.6–332.4)	2996.1 (2918.5–3075.3)	9.3 (9–9.5)	67 (66.6–67.4)	294 (269.7–319.9)	4.4 (4–4.8)
60–69 years	743.2 (741.6–744.7)	5989.3 (5886.3–6093.6)	8.5 (8.4–8.7)	164.2 (163.5–164.9)	594.5 (561.5–629)	3.6 (3.4–3.9)
70–79 years	1528.3 (1525.8–1530.8)	9204 (9080–9329.1)	6.5 (6.4–6.6)	417.7 (416.4–419)	1177.2 (1131.3–1224.5)	2.8 (2.7–2.9)
80–89 years	2609.5 (2604.9–2614.2)	10,929.1 (10,760.4–11,099.6)	4.6 (4.5–4.7)	901 (898.3–903.8)	1863.7 (1791–1938.5)	2.1 (2–2.2)
>89 years	3376.3 (3363.1–3389.5)	10,701.5 (10,283.2–11,130.9)	3.4 (3.3–3.6)	1366.5 (1358.1–1374.9)	2143.2 (1950.1–2349.9)	1.6 (1.5–1.7)
Total	362.9 (362.5–363.2)	5118.5 (5076.8–5160.4)	14.8 (14.7–14.9)	102 (101.8–102.2)	681.9 (666.4–697.7)	6.7 (6.6–6.8)

**Table 4:** Characteristics of lithiasis patients stratified by major adverse cardiovascular events. Demographics, cardiovascular risk factors, and mortality. Groups present statistical differences in all categories (*p* < 0.0001).

	No Events n (% CI95%)	Coronary Heart Disease	Stroke n (% CI95%)
n (Patients)	630,735	9352	2120
Gender (M/F) (%) (CI95%)	352,147 (55.8%) (55.7–55.9)	5674 (60.7%) (59.7–61.7)	1174 (55.4%) (53.2–57.5)
Mean Age (years) (CI95%)	57.3 (57.3–57.4)	62.7 (62.3–63)	66.2 (65.5–66.9)
Diabetes n (%) (CI95%)	25,365 (4%) (4–4)	288 (3.1%) (2.7–3.5)	117 (5.5%) (4.6–6.6)
Hypertension n (%) (CI95%))	126,538 (20.1%) (20–20.2)	2116 (22.6) (21.8–23.5)	593 (28%) (26.1–29.9)
Hyperlipidemia n (%) (CI95%)	42,184 (6.7%) (6.6–6.8)	818 (8.7%) (8.2–9.3)	180 (8.5%) (7.3–9.8)
Obesity n (%) (CI95%)	22,011 (3.5%) (3.4–3.5)	345 (3.7%) (3.3–4.1)	114(5.4%) (4.5–6.4)
Smoking n (%) (CI95%)	15,349 (2.4%) (2.4–2.5)	78 (0.8%) (0.7–1)	30 (1.4%) (1–2)
Mortality n (%) (CI95%)	28,195 (4.5%) (4.4–4.5)	1134 (12.1%) (11.5–12.8)	389 (18.3%) (16.7–20.1)

**Table 5:** Association between demographical and cardiovascular comorbidities and risk of cardiovascular or cerebrovascular events in patients with renal or lithiasis using a univariate analysis (hazard ratio). Results are given with 95% confidence intervals in brackets. ns: *p* > 0.05.

	Cardiovascular		Cerebrovascular	
	Hazard Ratio	*p*	Hazard Ratio	*p*
Gender (male/female)	0.81 (0.77–0.84)	*p* < 0.0001	1 (0.92–1.09)	ns
Diabetes (yes/no)	1.81 (1.61–2.03)	*p* < 0.0001	2.97 (2.46–3.6)	*p* < 0.0001
Hypertension (yes/no)	1.47 (1.40–1.54)	*p* < 0.0001	1.91 (1.74–2.10)	*p* < 0.0001
Hyperlipidemia (yes/no)	1.6 (1.49–1.72)	*p* < 0.0001	1.52 (1.30–1.77)	*p* < 0.0001
Overweight (yes/no)	1.45 (1.31–1.60)	*p* < 0.0001	2.05 (1.71–2.46)	*p* < 0.0001
Smoking (yes/no)	0.92 (0.74–1.15)	ns	1.34 (0.93–1.92)	ns

**Table 6:** Multivariate analysis describing the association between demographical and cardiovascular morbidities in the presence of all others for the risk of developing ischemic cardiac or cerebrovascular events in patients with kidney stones. (ns) non significant.

**Ischemic Heart Disease**	**Sig.**	**Hazard Ratio**	**95.0% CI for Hazard Ratio**	
			**Inferior**	**Superior**
Gender (M/F)	*p* < 0.0001	0.79	0.76	0.83
Diabetes	*p* < 0.0001	1.60	1.42	1.80
Hypertension	*p* < 0.0001	1.36	1.29	1.43
Hyperlipidemia	*p* < 0.0001	1.42	1.32	1.52
Overweight	*p* < 0.0001	1.25	1.12	1.38
Smoking	ns	0.82	0.66	1.03
**Cerebrovascular Disease**	**Sig.**	**Hazard Ratio**	**95.0% CI for Hazard Ratio**	
			**Inferior**	**Superior**
Gender	ns	0.98	0.90	1.06
Diabetes	*p* < 0.0001	2.33	1.92	2.83
Hypertension	*p* < 0.0001	1.70	1.54	1.89
Hyperlipidemia	*p* = 0.02	1.21	1.03	1.42
Overweight	*p* < 0.0001	1.56	1.29	1.88
Smoking	ns	1.09	0.75	1.57

## Data Availability

The datasets generated during and/or analyzed during the current study are available from the corresponding author upon reasonable request.

## References

[1] Cheungpasitporn W., Thongprayoon C., Mao M.A., O’Corragain O.A., Edmonds P.J., Erickson S.B. (2014). The Risk of Coronary Heart Disease in Patients with Kidney Stones: A Systematic Review and Meta-analysis. N. Am. J. Med. Sci..

[2] Peng J.P., Zheng H. (2017). Kidney stones may increase the risk of coronary heart disease and stroke: A PRISMA-Compliant meta-analysis. Medicine.

[3] Liu Y., Li S., Zeng Z., Wang J., Xie L., Li T., He Y., Qin X., Zhao J. (2014). Kidney stones and cardiovascular risk: A meta-analysis of cohort studies. Am. J. Kidney Dis..

[4] Muschialli L., Mannath A., Moochhala S.H., Shroff R., Ferraro P.M. (2024). Epidemiological and biological associations between cardiovascular disease and kidney stone formation: A systematic review and meta-analysis. Nutr. Metab. Cardiovasc. Dis..

[5] Ferraro P.M., Taylor E.N., Eisner B.H., Gambaro G., Rimm E.B., Mukamal K.J., Curhan G.C. (2013). History of kidney stones and the risk of coronary heart disease. JAMA.

[6] Sáenz-Medina J., Muñoz M., Sanchez A., Rodriguez C., Jorge E., Corbacho C., Izquierdo D., Santos M., Donoso E., Virumbrales E. (2020). Nox1-derived oxidative stress as a common pathogenic link between obesity and hyperoxaluria-related kidney injury. Urolithiasis.

[7] Prieto D., Contreras C., Sanchez A. (2014). Endothelial dysfunction, obesity and insulin resistance. Curr. Vasc. Pharmacol..

[8] Heitzer T., Schlinzig T., Krohn K., Meinertz T., Münzel T. (2001). Endothelial dysfunction, oxidative stress, and risk of cardiovascular events in patients with coronary artery disease. Circulation.

[9] Davignon J., Ganz P. (2004). Role of endothelial dysfunction in atherosclerosis. Circulation.

[10] Vanhoutte P.M., Shimokawa H., Feletou M., Tang E.H. (2017). Endothelial dysfunction and vascular disease—A 30th anniversary update. Acta Physiol..

[11] Saenz-Medina J., Muñoz M., Rodriguez C., Contreras C., Sánchez A., Coronado M.J., Ramil E., Santos M., Carballido J., Prieto D. (2022). Hyperoxaluria Induces Endothelial Dysfunction in Preglomerular Arteries: Involvement of Oxidative Stress. Cells.

[12] Instituto Nacional de Estadística (INE) Demography and Population. https://www.ine.es.

[13] BOE (2015). Real Decreto 69/2015, de 6 de Febrero, por el que se Regula el Registro de Actividad de Atención Sanitaria Especializada. https://boe.es.

[14] Jeong I.G., Kang T., Bang J.K., Park J., Kim W., Hwang S.S., Kim H.K., Park H.K. (2011). Association between metabolic syndrome and the presence of kidney stones in a screened population. Am. J. Kidney Dis..

[15] Taylor E.N., Stampfer M.J., Curhan G.C. (2005). Obesity, weight gain, and the risk of kidney stones. JAMA.

[16] West B., Luke A., Durazo-Arvizu R.A., Cao G., Shoham D., Kramer H. (2008). Metabolic syndrome and self-reported history of kidney stones: The National Health and Nutrition Examination Survey (NHANES III) 1988–1994. Am. J. Kidney Dis..

[17] Romero V., Akpinar H., Assimos D.G. (2010). Kidney stones: A global picture of prevalence, incidence, and associated risk factors. Rev. Urol..

[18] Ramello A., Vitale C., Marangella M. (2000). Epidemiology of nephrolithiasis. J. Nephrol..

[19] Umekawa T., Byer K., Uemura H., Khan S.R. (2005). Diphenyleneiodium (DPI) reduces oxalate ion- and calcium oxalate monohydrate and brushite crystal-induced upregulation of MCP-1 in NRK 52E cells. Nephrol. Dial. Transpl..

[20] Evan A.P., Worcester E.M., Coe F.L., Williams J., Lingeman J.E. (2015). Mechanisms of human kidney stone formation. Urolithiasis.

[21] Yencilek E., Sarı H., Yencilek F., Yeşil E., Aydın H. (2017). Systemic endothelial function measured by flow-mediated dilation is impaired in patients with urolithiasis. Urolithiasis.

[22] Vidavsky N., Kunitake J.A.M.R., Estroff L.A. (2021). Multiple Pathways for Pathological Calcification in the Human Body. Adv. Healthc. Mater..

[23] Yazici O., Narter F., Erbin A., Aydin K., Kafkasli A., Sarica K. (2019). Effect of endothelial dysfunction on the pathogenesis of urolithiasis in patients with metabolic syndrome. Aging Male.

[24] Sáenz-Medina J., Martinez M., Rosado S., Durán M., Prieto D., Carballido J. (2021). Urolithiasis Develops Endothelial Dysfunction as a Clinical Feature. Antioxidants.

[25] Ferreira-González I. (2014). The epidemiology of coronary heart disease. Rev. Esp. Cardiol..

[26] López-Bescós L., Cosín J., Elosua R., Cabadés A., Reyes M.D.L., Arós F., Diago J.L., Asín E., Castro-Beiras A., Marrugat J. (1999). The prevalence of angina and cardiovascular risk factors in the different autonomous communities of Spain: The PANES Study. Prevalencia de Angina en España. Rev. Esp. Cardiol..

[27] Nichols NT M., Luengo-Fernandez R., Leal J., Gray A., Scarborough P., Rayner Muropean Cardiovascular Disease Statistics 2012 (2012). European Heart Network, Brussels and European Society of Cardiology Sophia Antipolis. https://www.escardio.org/static-file/Escardio/Press-media/press-releases/2013/EU-cardiovascular-disease-statistics-2012.pdf.

[28] Xu M., Zhao Z., Shen F., Hu R., Lu J., Xu Y., Wang T., Li M., Chen G., Chen L. (2022). Modification effect of changes in cardiometabolic traits in association between kidney stones and cardiovascular events. Front. Cardiovasc. Med..

[29] Hsu C.-Y., Chen Y.-T., Huang P.-H., Leu H.-B., Su Y.-W., Chiang C.-H., Chen J.-W., Chen T.-J., Lin S.-J., Chan W.-L. (2016). The association between urinary calculi and increased risk of future cardiovascular events: A nationwide population-based study. J. Cardiol..

[30] Zhang C., Ni J., Chen Z. (2022). Apolipoprotein B Displays Superior Predictive Value Than Other Lipids for Long-Term Prognosis in Coronary Atherosclerosis Patients and Particular Subpopulations: A Retrospective Study. Clin. Ther..

[31] Kawel-Boehm N., Kronmal R., Eng J., Folsom A., Burke G., Carr J.J., Shea S., Lima J.A.C., Bluemke D.A., O’brien C. (2019). Left Ventricular Mass at MRI and Long-term Risk of Cardiovascular Events: The Multi-Ethnic Study of Atherosclerosis (MESA). Radiology.

[32] Torricelli F.C., De S.K., Gebreselassie S., Li I., Sarkissian C., Monga M. (2014). Dyslipidemia and kidney stone risk. J. Urol..

[33] Fuchs F.D., Whelton P.K. (2020). High Blood Pressure and Cardiovascular Disease. Hypertension.

[34] Taylor E.N., Stampfer M.J., Curhan G.C. (2005). Diabetes mellitus and the risk of nephrolithiasis. Kidney Int..

[35] Kim Y.J., Park M.S., Kim W.T., Yun S.J., Kim W.J., Lee S.C. (2011). Hypertension influences recurrent stone formation in nonobese stone formers. Urology.

[36] Khan S.R. (2012). Is oxidative stress, a link between nephrolithiasis and obesity, hypertension, diabetes, chronic kidney disease, metabolic syndrome. Urol. Res..

[37] Saenz-Medina J., Muñoz M., Rodriguez C., Sanchez A., Contreras C., Carballido-Rodríguez J., Prieto D. (2022). Endothelial Dysfunction: An Intermediate Clinical Feature between Urolithiasis and Cardiovascular Diseases. Int. J. Mol. Sci..

[38] Arias Fúnez F., García Cuerpo E., Lovaco Castellanos F., Escudero Barrilero A., Avila Padilla S., Villar Palasí J. (2000). Epidemiology of urinary lithiasis in our Unit. Clinical course in time and predictive factors. Arch. Esp. Urol..

